# Human lung fibroblast-to-myofibroblast transformation is not driven by an LDH5-dependent metabolic shift towards aerobic glycolysis

**DOI:** 10.1186/s12931-019-1058-2

**Published:** 2019-05-09

**Authors:** Eva Schruf, Victoria Schroeder, Christian A. Kuttruff, Sabine Weigle, Martin Krell, Maryke Benz, Tom Bretschneider, Alexander Holweg, Michael Schuler, Manfred Frick, Paul Nicklin, James P. Garnett, Mirko C. Sobotta

**Affiliations:** 1 Immunology & Respiratory Diseases Research, Boehringer Ingelheim Pharma GmbH & Co. KG, Birkendorfer Straße 65, 88397 Biberach an der Riss, Germany; 2 Medicinal Chemistry, Boehringer Ingelheim Pharma GmbH & Co. KG, Birkendorfer Straße 65, 88397 Biberach an der Riss, Germany; 3 Drug Discovery Sciences, Boehringer Ingelheim Pharma GmbH & Co. KG, Birkendorfer Straße 65, 88397 Biberach an der Riss, Germany; 4 Institute of General Physiology, University of Ulm, Ulm, Germany

**Keywords:** Idiopathic pulmonary fibrosis, Fibroblast-to-myofibroblast transformation, Metabolic shift, Aerobic glycolysis, Lactate dehydrogenase, TGF-β1, Human lung fibroblasts

## Abstract

**Background:**

Idiopathic pulmonary fibrosis (IPF) is a fatal respiratory disease characterized by aberrant fibroblast activation and progressive fibrotic remodelling of the lungs. Though the exact pathophysiological mechanisms of IPF remain unknown, TGF-β1 is thought to act as a main driver of the disease by mediating fibroblast-to-myofibroblast transformation (FMT). Recent reports have indicated that a metabolic shift towards aerobic glycolysis takes place during FMT and that metabolic shifts can directly influence aberrant cell function. This has led to the hypothesis that inhibition of lactate dehydrogenase 5 (LDH5), an enzyme responsible for converting pyruvate into lactate, could constitute a therapeutic concept for IPF.

**Methods:**

In this study, we investigated the potential link between aerobic glycolysis and FMT using a potent LDH5 inhibitor (Compound 408, Genentech). Seahorse analysis was performed to determine the effect of Compound 408 on TGF-β1-driven glycolysis in WI-38 fibroblasts. TGF-β1-mediated FMT was measured by quantifying α-smooth muscle actin (α-SMA) and fibronectin in primary human lung fibroblasts following treatment with Compound 408. Lactate and pyruvate levels in the cell culture supernatant were assessed by LC-MS/MS. In addition to pharmacological LDH5 inhibition, the effect of siRNA-mediated knockdown of LDHA and LDHB on FMT was examined.

**Results:**

We show that treatment of lung fibroblasts with Compound 408 efficiently inhibits LDH5 and attenuates the TGF-β1-mediated metabolic shift towards aerobic glycolysis. Additionally, we demonstrate that LDH5 inhibition has no significant effect on TGF-β1-mediated FMT in primary human lung fibroblasts by analysing α-SMA fibre formation and fibronectin expression.

**Conclusions:**

Our data strongly suggest that while LDH5 inhibition can prevent metabolic shifts in fibroblasts, it has no influence on FMT and therefore glycolytic dysregulation is unlikely to be the sole driver of FMT.

**Electronic supplementary material:**

The online version of this article (10.1186/s12931-019-1058-2) contains supplementary material, which is available to authorized users.

## Background

Idiopathic pulmonary fibrosis (IPF) is a chronic lung disease of unknown aetiology characterized by progressive fibrosis of the lung parenchyma and lung function decline [1]. Following the approval of Nintedanib and Pirfenidone, a reduction in the rate of disease progression can now be achieved in many patients, however IPF remains inevitably fatal and patients face a poor prognosis with a median survival time of only 2–3 years from the time of diagnosis [1–3].

While the exact pathophysiological mechanisms of IPF remain unknown, recent evidence suggests that various environmental exposures in combination with age-related and genetic predisposition result in a susceptibility to abnormal wound healing in response to repetitive alveolar epithelial cell micro-injuries [4–6]. According to the current view, the abnormally activated alveolar epithelium secretes mediators that stimulate uncontrolled fibroblast proliferation and excessive extracellular matrix formation in the lung interstitium, resulting in scarring and loss of lung function [6–8]. Among the variety of likely secreted mediators, transforming growth factor-beta (TGF-β) is considered to be the major cytokine that induces the exaggerated matrix deposition within the IPF lung, mainly through fibroblast recruitment and transformation [6, 9, 10]. Following activation by TGF-β or other pro-fibrotic stimuli, fibroblasts differentiate to myofibroblasts, which secrete excessive amounts of collagen-rich extracellular matrix and thereby constitute the primary pathologic fibroblast phenotype in IPF [11–13].

Recent reports have indicated that metabolic reprogramming during myofibroblast differentiation could play a role in the pathogenesis of IPF [14–18]. Aberrant cellular metabolism has been linked to a variety of human diseases. For instance, it has long been known that cancer cells undergo a glycolytic reprogramming under normoxic conditions [19]. A similar type of metabolic shift towards aerobic glycolysis has recently been suggested to play a role in fibrosis, after levels of lactic acid and glycolytic intermediate metabolites were found to be increased in IPF lungs [7, 15, 20]. Xie et al. have shown that lung fibroblasts demonstrate augmented glycolysis and an upregulation of glycolytic enzymes during TGF-β-induced myofibroblast differentiation in vitro and suggested glycolytic inhibition as a potential therapeutic approach in IPF [14]. In accordance, it has been reported that lactate dehydrogenase 5 (LDH5) might play a role in TGF-β-induced myofibroblast differentiation [15, 18].

Due to increasing evidence, that metabolic reprogramming and elevated lactic acid concentrations might play an important role in the pathogenesis of IPF, pharmacologic LDH5 inhibition has emerged as a potential strategy to inhibit myofibroblast differentiation in IPF. Recently, Kottmann et al. have reported that the natural nonselective LDH inhibitor Gossypol as well as genetic knockdown of LDHA (gene name of protein subunit of LDH5) expression could inhibit TGF-β-induced myofibroblast differentiation in human lung fibroblasts [21]. However, Gossypol, which can be extracted from the pigment glands of the cotton plant and has also been investigated as a potential anti-cancer drug, has been shown to demonstrate high unspecific cytotoxic and genotoxic effects in a multitude of mammalian cell types due to its structural characteristics [22–29].

In the last couple of years, there has been a substantial increase in publications and filed patents describing small molecule LDH inhibitors from both pharma companies and academic groups reflecting the ongoing interest in this target [24, 30, 31]. In spite of this progress regarding the number of available LDH inhibitors, many compounds still lack an optimal alignment of cell activity, selectivity and acceptable physicochemical properties. The only LDH inhibitor that was so far reported to be capable of modulating LDHA activity both in vitro and in vivo was recently published by Genentech [32]*.*

In this study, we aimed to investigate the potential link between aerobic glycolysis and myofibroblast differentiation using Tool Compound 408, a potent LDH5 inhibitor that was previously described in a Genentech patent (WO 2015/140133 A1).

## Methods

### Synthesis of compound 408

LDH5 inhibitor Compound 408 was synthesized according to the procedures described in patent WO 2015/140133 A1. Purification of Compound 408: The final crude compound was purified by silica gel column chromatography (eluent: MeOH:dichloromethane = 1:100) to provide compound 408 as a colourless solid. For details see Additional file 1 :Figure S1 .

### NHLF culture conditions

Primary human lung fibroblasts (Lonza, Walkersville, MD, USA) were grown in fibroblast basal medium (FBM) (CC-3131, Lonza Walkersville, Inc., Walkersville, MD, USA) supplemented with FGM-2 SingleQuot Kit Supplements & Growth Factors (CC-4126, Lonza) at 37 °C and 5% CO_2_. Cells were passaged maximum 10 times before use.

### NHLF α-SMA Western blot replacement assay and cytotoxicity assay

4000 primary human lung fibroblasts were seeded per well for α-SMA Western blot replacement and cytotoxicity assays. Determination of α-SMA expression was performed as previously described [33]. In brief, cells were starved in serum-free DMEM for 24 h, pre-treated with different concentrations of Compound 408 for 20 min and stimulated with TGF-β1 (5 ng/ mL) for 48 h. Lysates were assayed in a Western blot replacement assay [Meso Scale Discovery (MSD), Rockville, MD, USA]. An MSD sector imager (MSD) was used for analysis. The percentage of α-SMA expression was calculated relative to the control. Cytotoxicity was measured by ELISA using the LDH Cytotoxicity Detection Kit (Roche) according to the manufacturer’s instructions and by CyQuant Direct Cell Proliferation Assay.

### FMT quantification by visual α-SMA and fibronectin analysis

FMT quantification was carried out similarly as previously described [34]. Primary human lung fibroblasts were plated in a poly-D-lysine coated 384 CellCarrier microtiter plate from PerkinElmer in fibroblast basal medium (FBM) with FGM-2TM Single Quots (Lonza) at a density of 1000 or 2000 cells per well for compound or siRNA testing, respectively. After 24 h, the medium was replaced by the same medium containing no (compound) and 0.1% (siRNA) foetal calf serum (starvation medium). 48 h after cell seeding fibroblast to myofibroblast differentiation was initiated by replacing the starvation medium with starvation medium containing a mixture of Ficoll 70 and 400 (GE Healthcare; 37.5 mg/mL and 25 mg/ mL, respectively), 200 μM vitamin C and 5 ng/ mL TGF-β1 [35]. Compounds were added 30 min before the addition of TGF-β1 in medium containing 0.1% DMSO. After 72 h the cell culture medium was removed and cells were fixed with 100% ice-cold methanol for 30 min. Next, cells were washed with PBS, permeabilised for 20 min using 1% Triton-X-100 (Sigma), washed and blocked for 30 min with 3% BSA in PBS. After an additional wash step, cell nuclei were stained using 1 μM Hoechst 33342 (Molecular Probes) and alpha smooth muscle actin (α-SMA) and collagen I were stained using monoclonal antibodies (Sigma: A2547 and SAB4200678, each 1:1000 diluted). For detection of primary antibodies, cells were washed and incubated for 30 min at 37 °C with AF647-goat-anti-mouse IgG2b (α-SMA) and AF568goat-anti-mouse IgG1 (collagen I) antibodies. After removal of secondary antibodies, cells were stained with HCS Cell Mask Green stain (Invitrogen, 1:50000). Following a final wash step, images were acquired in a GE Healthcare InCell 2200 Analyzer, using 2D-Deconvolution for nuclei (Hoechst channel), cells (FITC channel), α-SMA (Cy5 channel) and collagen I (TexasRed channel), and images were transferred to Perkin Elmer’s Columbus Image Storage and Analysis system.

Image analysis was performed similar to a previously published analysis image protocol based on the IN Cell Developer Toolbox 1.9.1. [34] Briefly, using the building blocks of Perkin Elmer’s Columbus Image Analysis system, first nuclei acquired with the Hoechst channel were detected using the building block (BB) “nuclei”. Second, cells were defined with the BB “find cytoplasm” from the FITC channel. α-SMA fibres and collagen I area were defined by two individual BBs “find simple image region” based on images acquired in the Cy5 and TexasRed channels, respectively. Both α-SMA fibers and collagen I readouts were normalized to the number of cells per image field. Total number of cells, number of fibre-like α-SMA structures/cell and total collagen area/total number of cells were used as parameters to quantify effects of siRNAs and compounds.

### Analysis of lactate and pyruvate by LC-MS/MS

Primary human lung fibroblasts were cultured and stimulated with 5 ng/ mL TGF-β1 and different concentrations of Compound 408 as described above in the FMT quantification assay. 72 h after TGF-β1 treatment the culture medium was collected and 10 μL cell supernatant was diluted in 190 μL acetonitrile and centrifuged at 18000 g for 10 min at 4 °C. For quantification purposes an external calibration was prepared in acetonitrile for lactate (R2 = 0.998) and pyruvate (R2 = 0.999). Both lactate and pyruvate levels from cell culture supernatants were determined by liquid chromatography-tandem mass spectrometry (LC-MS/MS) system. An Agilent 1290 Infinity II Multisampler equipped with an Agilent 1290 Infinity II High Speed Pump (Waldbronn, Germany) was coupled with an ABSciex 6500+ Triple Quad mass spectrometer (MS) (Darmstadt, Germany). Instrument control, data acquisition and processing were performed using the Analyst 1.6.3 software.

A method described by Zhang et al. was applied and modified. [36] The LC-MS/MS system was equipped with an ACQUITY UPLC BEH Amide column (2.1 × 150 mm, 1.7 μm), eluent A (50% acetonitrile/50% H2O) and eluent B (95% acetonitrile/5% H2O), both supplemented with 20 mM ammonium acetate and 20 mM ammonium hydroxide (pH 9) in the aqueous phase respectively. 5 μL of sample was injected at a flowrate of 0.4 mL/ min and a column temperature of 60 °C. The analytes were separated within a 8 min gradient as following: 0–1 min 100% eluent B; 1–2 min 100–70% eluent B; 2–3.5 min 70% eluent B; 3.5–4.5 min 70–0% eluent B; 4.5–5.5 min 0% eluent B; 5.5–6 min 0–100% eluent B and a reconditioning at 100% eluent B for 2 min. The Triple Quad MS was operated in negative electrospray mode acquiring the following transitions: lactate quantifier 89.0/43.0 at DP = − 50 and CE = − 15; lactate qualifier 89.0/71.0 at DP = − 50 and CE = − 15 and the pyruvate quantifier 86.8/42.9 at DP = − 50 and CE = − 15. Ion source temperature was set at 500 °C, the ion spray voltage at − 4.5 kV, ion source gas 1 and ion source gas 2 both at 50 psi and the curtain gas at 40 psi. All chemicals and references were purchased from Sigma-Aldrich (Taufkirchen, Germany).

### Seahorse analysis

Eight thousand WI-38 cells (passaged not more than 10 times after thawing) per well were seeded into a Seahorse XF96 plate (EMEM, 10% heat-inactivated FBS, 1% MEM non-essential amino acid solution, 1% GlutaMAX-I) and left for 1 h at room temperature before incubation at 37 °C, 5% CO_2_ for 24 h. After visual inspection by light microscopy, cells were washed twice with starvation medium (EMEM) and kept in starvation medium at 37 °C, 5% CO_2_ for 24 h. After starvation, cells were treated with fresh starvation medium with or without 5 ng/ mL TGF-β1 and compound or vehicle (DMSO). A symmetrical plate layout was used that minimizes edge effects and optimizes assay robustness (Additional file 1: Figure S2). The edge wells were excluded for data analysis. For analysis of glycolysis parameters, Seahorse assays were performed according to the manufacturer’s instructions of the Seahorse Bioscience XF cell glycolysis stress test kit (#103020–100). Assays were performed in the presence of compounds (vehicle DMSO) and after visual inspection of the cells by light microscopy. During the assay cells were treated with 10 mM Glucose, 1.25 μM Oligomycin and 50 mM 2-Deoxyglucose. Mixing time was 3 min and measure time 4 min over a period of 3 cycles each.

## Results

### TGF-β1 induces a metabolic shift in human lung fibroblasts towards higher energy expenditure

**Fig. 1:**
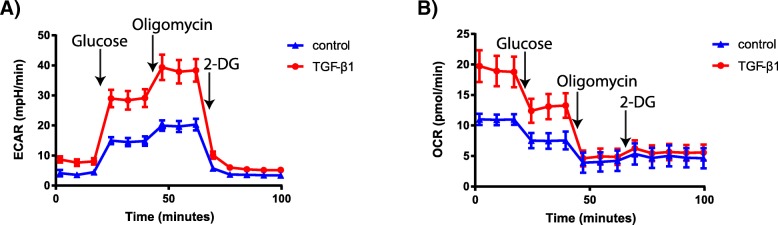
TGF-β1 increases glycolysis and oxygen consumption in human lung fibroblasts. Extracellular acidification rate (ECAR, panel **a**) and oxygen consumption rate (OCR, panel **b**) of WI-38 fibroblasts, with and without 24 h pre-treatment with 5 ng/ mL TGF-β1. During the glycolysis stress test assay cells were treated with 10 mM Glucose, 1.25 μM Oligomycin and 50 mM 2-Deoxyglucose (2-DG). *N* = 3

To confirm previous observations that TGF-β signalling augments glycolysis in human lung fibroblasts, we performed Seahorse analysis of WI-38 human lung fibroblasts. After starvation, WI-38 cells were treated for 24 h with 5 ng/ mL TGF-β1 and the extracellular acidification rate and the oxygen consumption rate were measured. TGF-β1 treatment of WI-38 lung fibroblasts resulted in increased glycolysis (glucose-induced increase in extracellular acidification rate; ECAR) and glycolytic capacity (Oligomycin-induced ECAR), consistent with enhanced aerobic glycolysis (Fig. 1a). This corresponded with elevated oxygen consumption under non-glycolytic (prior to glucose addition) and glycolytic conditions, indicative of enhanced overall energy expenditure (Fig. 1b).

### An LDH5 inhibitor (Compound 408) reduces basal and TGF-β1-driven glycolysis

**Fig. 2:**
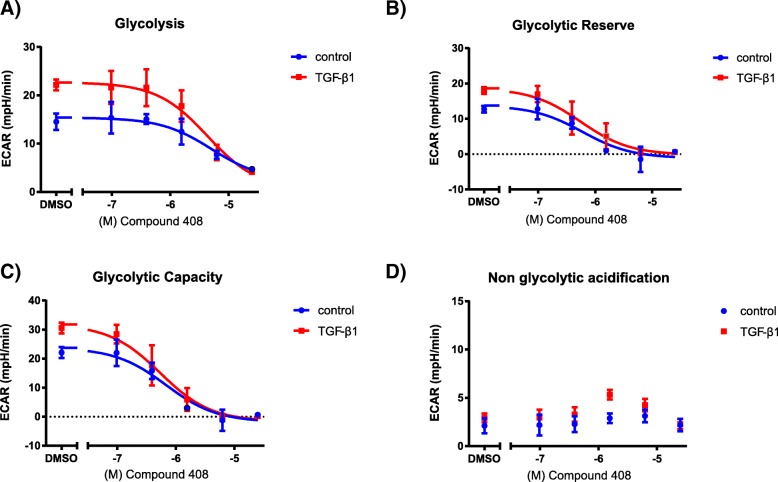
Compound 408 inhibits glycolysis without influencing non-glycolytic acidification in human lung fibroblasts. Glycolysis (**a**), glycolytic reserve (**b**), glycolytic capacity (**c**) and non-glycolytic acidification (**d**) were determined in WI-38 fibroblasts pre-treated for 24 h with LDH5 inhibitor (Compound 408) and TGF-β1, based on changes in ECAR with 10 mM Glucose, 1.25 μM Oligomycin and 50 mM 2-Deoxyglucose (2-DG) as depicted in Fig. 1a. *N* = 3

Subsequently we aimed to determine the effect of a previously described LDH5 inhibitor, Compound 408 (Genentech), on basal as well as TGF-β1-driven glycolysis in lung fibroblasts. For this, WI-38 fibroblasts were simultaneously stimulated with 5 ng/mL TGF-β1 and rising concentrations of Compound 408 for 24 h before the cells were analysed by Seahorse. As expected, Compound 408 dose-dependently inhibited glycolysis, glycolytic capacity and glycolytic reserve in untreated and TGF-β1-treated WI-38 lung fibroblasts, with a mean IC50 of 0.6 μM for the glycolytic capacity and 4.5 μM for glycolysis for TGF-β1-treated cells (Fig. 2a, b, c; Additional file 1 :Figure S3). To exclude glycolysis-independent effects of Compound 408, non-glycolytic acidification (ECAR prior to addition of glucose) was measured and no significant changes were observed (Fig. 2d). To assess whether Compound 408 negatively affects cell viability, we performed a CyQuant Direct Cell Proliferation Assay and a LDH Cytotoxicity Detection ELISA in normal human primary human lung fibroblasts. No dose-dependent decrease in cell viability was detected after 48 h of treatment, indicating that Compound 408 does not decrease cell viability (Additional file 1 :Figure S4a, b).

### LDH5 inhibition does not inhibit fibroblast-to-myofibroblast transformation (FMT)

**Fig. 3:**
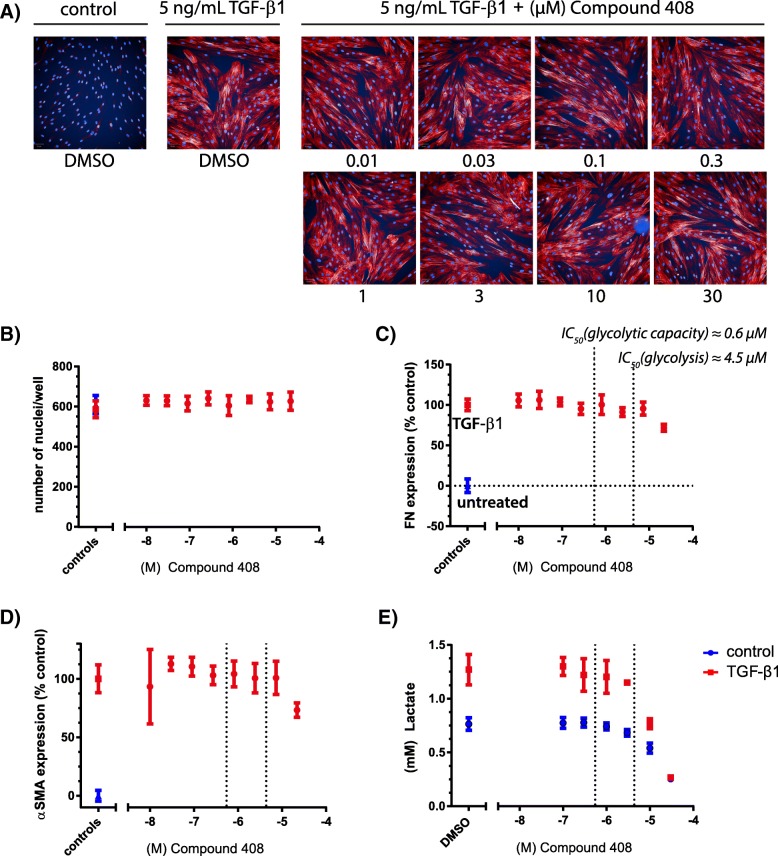
Inhibition of LDH5 by Compound 408 does not inhibit FMT in primary human lung fibroblasts. Image based analysis of α-SMA fibres, fibronectin (FN) and the numbers of nuclei in primary human lung fibroblasts treated with LDH5 inhibitor (Compound 408) and TGF-β1 for 72 h. Representative images of stained α-SMA fibres (**a**). Dose-response curves of the effect of increasing concentrations of LDH5 inhibitor (Compound 408) on nuclei numbers (**b**), fibronectin (FN) expression relative to untreated and TGF-β1 controls (**c**), α-SMA expression relative to untreated and TGF-β1 controls (**d**), and lactate production (**e**) with dashed lines indicating the mean IC50 for glycolytic capacity and glycolysis in TGF-β1-treated cells. *N* = 2, *n* = 8

We next sought to test the hypothesis that inhibition of LDH5 decreases fibroblast-to-myofibroblast transformation (FMT) [14–17]. NHLFs were treated with 5 ng/ mL TGF-β1 in the presence of Compound 408 and FMT was measured utilizing a semi-automated high-content analysis script by quantifying the number of α-smooth muscle actin (α-SMA) fibrils per fibroblast following immuno-fluorescent α-SMA staining, as well as by quantifying fibronectin staining [34, 35]. Representative images of stained α-SMA fibres in primary human lung fibroblasts following 72 h stimulation with TGF-β1 and different concentrations of Compound 408 are shown in Fig. 3a. The number of cell nuclei was not affected by Compound 408 treatment, even at high concentrations, indicating no impairment of cell viability (Fig. 3b). Compound 408 failed to decrease fibronectin and α-SMA expression in primary human lung fibroblasts, demonstrating that selective LDH5 inhibition does not impact FMT (Fig. 3c, d). Repetition of the primary human lung fibroblasts FMT assay with lower concentrations of TGF-β1 (2 ng/ mL and 1 ng/mL) showed similar results (Additional file 1 :Figure S5).

In addition to visually measuring FMT as described above, we measured lactate secretion in primary human lung fibroblasts cell culture media following stimulation with TGF-β1 and different concentrations of Compound 408 for 72 h to verify whether inhibition of lactate production by Compound 408 in primary human lung fibroblasts is maintained after prolonged stimulation periods. Lactate measurements revealed that Compound 408 dose-dependently inhibited TGF-β1 mediated lactate production in primary human lung fibroblasts efficiently throughout the 72 h stimulation, while also increasing pyruvate concentration (Fig. 3e; Additional file 1 :Figure S6).

To further validate our findings utilizing an alternative FMT quantification method, we performed α-SMA MSD ELISA in primary human lung fibroblasts treated with TGF-β1 and rising concentrations of Compound 408 for 48 h (Additional file 1 : Figure S4C). In line with our previous results, Compound 408 failed to decrease α-SMA protein expression in primary human lung fibroblasts.

Previously it has been shown that the naturally occurring compound Gossypol and its derivatives can inhibit glycolysis and FMT [21, 24]. We therefore tested the effect of the Gossypol as an alternative LDH5 inhibitor on FMT in primary human lung fibroblasts utilizing the aforementioned visual semi-automated high-content FMT assay. While Gossypol treatment dose-dependently reduced the detected α-SMA, this was accompanied by a decrease in the number of nuclei with rising concentrations of Gossypol, an effect we did not observe for Compound 408 even at high concentrations (Additional file 1: Figure S7). Our data suggest that while inhibition of glycolysis in itself is not cytotoxic, the apparent anti-fibrotic effects of Gossypol could, at least in part, be explained by its cytotoxicity in our experimental setting.

### siRNA-mediated LDHA knockdown has no inhibitory effect on FMT

**Fig. 4:**
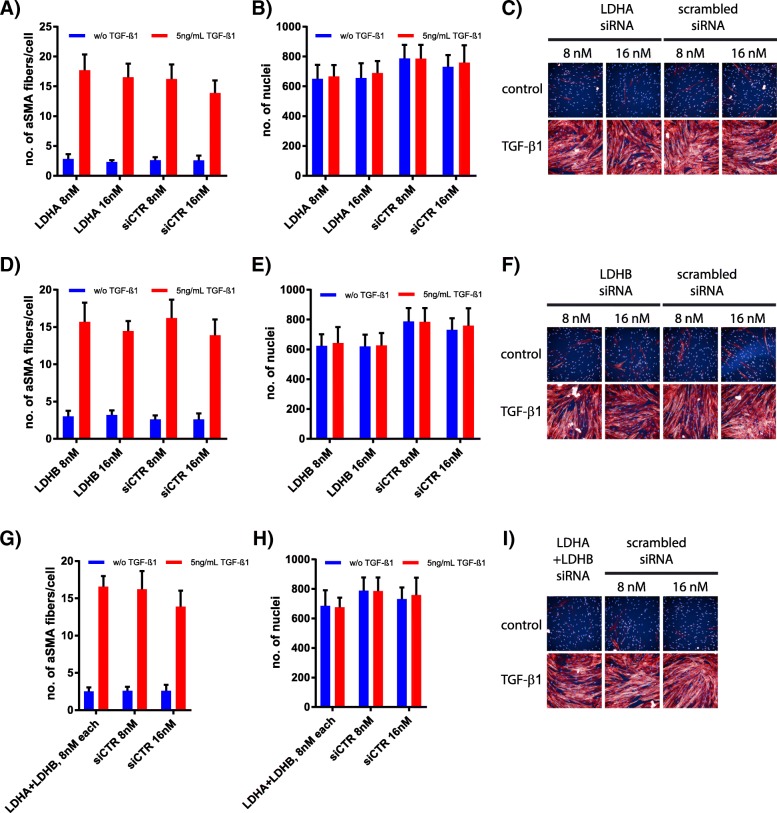
LDHA and LDHB knockdown does not inhibit FMT in primary human lung fibroblasts. α-SMA fibres per cell, nuclei numbers and representative images used for analysis of LDHA knockdown (**a**,**b**,**c**), LDHB knockdown (**d**,**e**,**f**) or dual LDHA/LDHB knockdown (**g**,**h**,**i**), in primary human lung fibroblasts after 72 h TGF-β1 stimulation. *N* = 2, *n* = 8

In an alternative approach, we investigated the effect of LDH inhibition on FMT in primary human lung fibroblasts by inhibiting LDHA and LDHB expression through siRNA treatment. Primary human lung fibroblasts were treated with siRNAs against LDHA and LDHB followed by TGF-β1 stimulation. After 72 h α-SMA fibres and the numbers of nuclei were visually analysed. Knockdown efficiency was confirmed by qRT-PCR (Additional file 1 :Figure S8). Similar to the limited influence of Compound 408, siRNA-mediated knockdown of LDHA (gene name of protein subunit of LDH5) in primary human lung fibroblasts had no effect on TGF-β1-induced FMT (Fig. 4a, c). Although LDH5 is reported to be the predominant isoenzyme involved in the production of lactate from pyruvate, we also investigated the contribution of LDHB. Knockdown of LDHB also failed to inhibit FMT (Fig. 4d, f). Additionally, combined knockdown of both LDH subunits produced no significant change in α-SMA fibre formation (Fig. 4g, i). Cell viability remained unaffected by all knockdown approaches, as evidenced by consistency in cell nuclei numbers (Fig. 4b, e, h).

## Discussion

In this study, we investigated the potential link between aerobic glycolysis and myofibroblast differentiation in human lung fibroblasts using the LDH5 inhibitor Compound 408. We found that selective LDH5 inhibition in primary human lung fibroblasts attenuates metabolic shifts towards aerobic glycolysis upon TGF-β1 stimulation but has no effect on FMT. We performed Seahorse analysis in human lung fibroblasts and, in accordance with previous publications, we observed that TGF-β1-induced myofibroblast differentiation is accompanied by an increase in glycolysis and oxygen consumption under non-glycolytic and glycolytic conditions [14, 16, 17]. We next investigated the potential causality between aerobic glycolysis and myofibroblast differentiation. The results of our Seahorse and FMT analyses show that the novel LDH5-inhibiting Compound 408 dose-dependently reduces basal and TGF-β1-driven glycolysis in human lung fibroblasts without negatively affecting cell viability. Quantification of FMT by visual α-SMA and fibronectin analysis in primary human lung fibroblasts following 72 h of 5 ng/ mL TGF-β1 stimulation reveals that neither Compound 408 nor siRNA-mediated knockdown of LDHA/ LDHB has an inhibitory effect on TGF-β1-mediated FMT. Stimulation of primary human lung fibroblasts with lower doses of TGF-β1 (2 ng/ mL and 1 ng/ mL) also failed to show efficacy of Compound 408 in the FMT assay. We further confirm that Compound 408 efficiently reduces the lactate concentration and increases the pyruvate concentration in primary human lung fibroblasts cell culture supernatant in the experimental setting that was applied in the FMT assay. We validated our findings by FMT quantification by α-SMA MSD ELISA in primary human lung fibroblasts treated with TGF-β1 and rising concentrations of Compound 408 for 48 h and observed no inhibitory effect of Compound 408 on FMT.

The aforementioned results indicate that shifts in metabolism towards aerobic glycolysis alone do not appear to be a significant stimulus for FMT. Our findings thereby contradict the current hypothesis in the field that myofibroblast differentiation in IPF is driven by glycolytic dysregulation and that LDH inhibition may represent a potential therapeutic approach for the disease [14–16]. However, this does not rule out the anti-fibrotic potential of LDH5 inhibition in other cell types, as highlighted by the ability of compound 408 to reduce tumor cell growth and proliferation [32], which may suggest a wider role in cellular proliferation and aberrant wound-healing that is associated with IPF. Furthermore, LDH5-dependent processes in other cells types may indirectly contribute to the myofibroblast pool through pro-fibrotic secretions or directly via the cell transformation such as epithelial-mesenchymal transition, as seen in cancer studies [37–40].

Previous reports stated that Gossypol, a natural nonselective LDH inhibitor, decreased TGF-β-induced FMT in primary human lung fibroblasts and radiation-induced pulmonary fibrosis in mice [21, 41]. However, Gossypol has long been known to influence a broad range of cellular functions due to its chemical structure and to have profound cytotoxic side effects [23, 24]. In their recent study, Kottmann et al. described a significant decrease of TGF-β-induced FMT in fibroblasts treated with 5 μM or 10 μM Gossypol and attributed this effect to Gossypol mediated LDH5 inhibition. In our study, we observed a severe decrease of primary human lung fibroblasts cell viability at 10 μM Gossypol, consistent with previous observations in WI-38 human embryonic lung fibroblasts [21, 25]. This indicates that the previously described decrease in FMT markers after Gossypol treatment may not be a direct effect of LDH5 inhibition.

It has been suggested in the literature that Gossypol might also exert its apparent anti-fibrotic effects through the inhibition of other LDH isoforms. To exclude a contribution of other isoforms, we performed siRNA-mediated knockdown in primary human lung fibroblasts and showed that neither LDHB-, nor LDHA-depletion, nor a combined knockdown of both genes had any significant inhibitory effect on TGF-β1-induced FMT. In consideration of these observations, it is possible that the previously described anti-fibrotic effects of the natural compound Gossypol might be attributed to an alternative LDH-unrelated mechanism of action.

## Conclusions

In this study we show for the first time, that LDH5 inhibition in human lung fibroblasts by a potent small molecule inhibitor (Compound 408) reduces aerobic glycolysis and lactate production, but does not affect TGF-β1-mediated fibroblast-to-myofibroblast differentiation. Based on our findings, we conclude that a LDH5-dependent metabolic shift towards aerobic glycolysis alone is not the key driver of FMT in human lung fibroblasts as we failed to observe a direct link between glycolytic regulation and TGF-β1-mediated FMT. Thus, we assume that an LDH5-dependent shift in cell metabolism is unlikely to be solely responsible for myofibroblast differentiation in the fibrotic lung. The results of our study add an important aspect to our understanding of the myofibroblast phenotype in the fibrotic lung and imply that further exploration of metabolic dysregulation in IPF beyond changes in glycolysis should be performed for more promising treatment strategies [42]. Our observations will thereby have implications on the design of future studies aiming to develop novel therapeutic concepts for IPF.

## Additional file

Additional file 1: Supplementary Information (Supplementary Figures S1–S8). (DOCX 2671 kb)

## Abbreviations

ECAR: Extracellular Acidification Rate
FBM: Fibroblast Basal Medium
FMT: Fibroblast-to-Myofibroblast Transformation
IPF: Idiopathic Pulmonary Fibrosis
LC-MS/MS: Liquid Chromatography-tandem Mass Spectrometry
LDH5: Lactate Dehydrogenase 5
TGF-β: Transforming Growth Factor-beta
α-SMA: alpha Smooth Muscle Actin

## References

[1] Raghu G, Collard HR, Egan JJ, Martinez FJ, Behr J, Brown KK (2011). An official ATS/ERS/JRS/ALAT statement: idiopathic pulmonary fibrosis: evidence-based guidelines for diagnosis and management. Am J Respir Crit Care Med.

[2] Hutchinson J, Fogarty A, Hubbard R, McKeever T (2015). Global incidence and mortality of idiopathic pulmonary fibrosis: a systematic review. Eur Respir J.

[3] Canestaro WJ, Forrester SH, Raghu G, Ho L, Devine BE (2016). Drug treatment of idiopathic pulmonary fibrosis: systematic review and network meta-analysis. Chest..

[4] Ryu JH, Moua T, Daniels CE, Hartman TE, Yi ES, Utz JP (2014). Idiopathic pulmonary fibrosis: evolving concepts. Mayo Clin Proc.

[5] Daccord C, Maher TM (2016). Recent advances in understanding idiopathic pulmonary fibrosis. F1000Research..

[6] King Talmadge E, Pardo Annie, Selman Moisés (2011). Idiopathic pulmonary fibrosis. The Lancet.

[7] Kang YP, Lee SB, Lee JM, Kim HM, Hong JY, Lee WJ (2016). Metabolic profiling regarding pathogenesis of idiopathic pulmonary fibrosis. J Proteome Res.

[8] Selman M, Pardo A (2002). Idiopathic pulmonary fibrosis: an epithelial/fibroblastic cross-talk disorder. Respir Res.

[9] Selman M, Pardo A (2014). Revealing the pathogenic and aging-related mechanisms of the enigmatic idiopathic pulmonary fibrosis. An integral model. Am J Respir Crit Care Med.

[10] Wuyts WA, Agostini C, Antoniou KM, Bouros D, Chambers RC, Cottin V (2013). The pathogenesis of pulmonary fibrosis: a moving target. Eur Respir J.

[11] Wolters PJ, Collard HR, Jones KD (2014). Pathogenesis of idiopathic pulmonary fibrosis. Annu Rev Pathol.

[12] Scotton CJ, Chambers RC (2007). Molecular targets in pulmonary fibrosis: the myofibroblast in focus. Chest..

[13] Hinz B, Phan SH, Thannickal VJ, Galli A, Bochaton-Piallat ML, Gabbiani G (2007). The myofibroblast: one function, multiple origins. Am J Pathol.

[14] Xie N, Tan Z, Banerjee S, Cui H, Ge J, Liu RM (2015). Glycolytic reprogramming in Myofibroblast differentiation and lung fibrosis. Am J Respir Crit Care Med.

[15] Kottmann RM, Kulkarni AA, Smolnycki KA, Lyda E, Dahanayake T, Salibi R (2012). Lactic acid is elevated in idiopathic pulmonary fibrosis and induces myofibroblast differentiation via pH-dependent activation of transforming growth factor-beta. Am J Respir Crit Care Med.

[16] Bernard K, Logsdon NJ, Ravi S, Xie N, Persons BP, Rangarajan S (2015). Metabolic reprogramming is required for Myofibroblast contractility and differentiation. J Biol Chem.

[17] Selvarajah B, Azuelos I, Forty E, Plate M, Anastasiou D, Mercer P, et al. Metabolic shift during TGF-β induced collagen synthesis. QJM: An International Journal of Medicine. 2016;109(suppl_1):S3-S.

[18] Judge JL, Nagel DJ, Owens KM, Rackow A, Phipps RP, Sime PJ (2018). Prevention and treatment of bleomycin-induced pulmonary fibrosis with the lactate dehydrogenase inhibitor gossypol. PLoS One.

[19] Li C, Zhang G, Zhao L, Ma Z, Chen H (2016). Metabolic reprogramming in cancer cells: glycolysis, glutaminolysis, and Bcl-2 proteins as novel therapeutic targets for cancer. World Journal of Surgical Oncology.

[20] Zhao YD, Yin L, Archer S, Lu C, Zhao G, Yao Y (2017). Metabolic heterogeneity of idiopathic pulmonary fibrosis: a metabolomic study. BMJ Open Respiratory Research.

[21] Kottmann RM, Trawick E, Judge JL, Wahl LA, Epa AP, Owens KM (2015). Pharmacologic inhibition of lactate production prevents myofibroblast differentiation. Am J Physiol Lung Cell Mol Physiol.

[22] Jaroszewski JW, Strom-Hansen T, Hansen SH, Thastrup O, Kofod H (1992). On the botanical distribution of chiral forms of gossypol. Planta Med.

[23] Jaroszewski JW, Kaplan O, Cohen JS (1990). Action of gossypol and rhodamine 123 on wild type and multidrug-resistant MCF-7 human breast cancer cells: 31P nuclear magnetic resonance and toxicity studies. Cancer Res.

[24] Granchi C, Paterni I, Rani R, Minutolo F (2013). Small-molecule inhibitors of human LDH5. Future Med Chem.

[25] Tuszynski GP, Cossu G. Differential cytotoxic effect of gossypol on human melanoma, colon carcinoma, and other tissue culture cell lines. Cancer Resaearch. 1984;44(2). 6581864

[26] Lin QR, Li CG, Zha QB, Xu LH, Pan H, Zhao GX (2016). Gossypol induces pyroptosis in mouse macrophages via a non-canonical inflammasome pathway. Toxicol Appl Pharmacol.

[27] Wang X, Wang J, Wong SC, Chow LS, Nicholls JM, Wong YC (2000). Cytotoxic effect of gossypol on colon carcinoma cells. Life Sci.

[28] Sahin F, Avci CB, Gunduz C, Sezgin C, Simsir IY, Saydam G (2010). Gossypol exerts its cytotoxic effect on HL-60 leukemic cell line via decreasing activity of protein phosphatase 2A and interacting with human telomerase reverse transcriptase activity. Hematology (Amsterdam, Netherlands).

[29] Rao MV, Narechania MB (2016). The genotoxic effects of anti-cancer drug gossypol on human lymphocytes and its mitigation by melatonin. Drug Chem Toxicol.

[30] Rani R, Kumar V (2016). Recent update on human lactate dehydrogenase enzyme 5 (hLDH5) inhibitors: a promising approach for Cancer chemotherapy. J Med Chem.

[31] Di Stefano G, Manerba M, Di Ianni L, Fiume L (2016). Lactate dehydrogenase inhibition: exploring possible applications beyond cancer treatment. Future Med Chem.

[32] Boudreau A, Purkey HE, Hitz A, Robarge K, Peterson D, Labadie S (2016). Metabolic plasticity underpins innate and acquired resistance to LDHA inhibition. Nat Chem Biol.

[33] Herrmann FE, Wollin L, Wirth J, Gantner F, Lammle B, Wex E (2017). Olodaterol shows anti-fibrotic efficacy in in vitro and in vivo models of pulmonary fibrosis. Br J Pharmacol.

[34] Aumiller V, Strobel B, Romeike M, Schuler M, Stierstorfer BE, Kreuz S (2017). Comparative analysis of lysyl oxidase (like) family members in pulmonary fibrosis. Sci Rep.

[35] Chen CZ, Peng YX, Wang ZB, Fish PV, Kaar JL, Koepsel RR (2009). The scar-in-a-jar: studying potential antifibrotic compounds from the epigenetic to extracellular level in a single well. Br J Pharmacol.

[36] Zhang W, Guo C, Jiang K, Ying M, Hu X (2017). Quantification of lactate from various metabolic pathways and quantification issues of lactate isotopologues and isotopmers. Sci Rep.

[37] Pavlides S, Whitaker-Menezes D, Castello-Cros R, Flomenberg N, Witkiewicz AK, Frank PG (2009). The reverse Warburg effect: aerobic glycolysis in cancer associated fibroblasts and the tumor stroma. Cell cycle (Georgetown, Tex).

[38] Koukourakis MI, Kalamida D, Mitrakas AG, Liousia M, Pouliliou S, Sivridis E (2017). Metabolic cooperation between co-cultured lung cancer cells and lung fibroblasts. Laboratory investigation; a journal of technical methods and pathology.

[39] Zhang Y, Lin S, Chen Y, Yang F, Liu S (2018). LDH-Apromotes epithelial-mesenchymal transition by upregulating ZEB2 in intestinal-type gastric cancer. OncoTargets and therapy.

[40] Zhao J, Huang X, Xu Z, Dai J, He H, Zhu Y (2017). LDHA promotes tumor metastasis by facilitating epithelialmesenchymal transition in renal cell carcinoma. Mol Med Rep.

[41] Judge JL, Lacy SH, Ku WY, Owens KM, Hernady E, Thatcher TH (2017). The lactate dehydrogenase inhibitor gossypol inhibits radiation-induced pulmonary fibrosis. Radiat Res.

[42] Zhao H, Dennery PA, Yao H. Metabolic reprogramming in the pathogenesis of chronic lung diseases including BPD, COPD, and pulmonary fibrosis. Am J Physiol Lung Cell Mol Physiol. 2018. 10.1152/ajplung.00521.2017 PMC5966782 29351437

