# Supplementary material for: Human lung fibroblast-to-myofibroblast transformation is not driven by an LDH5-dependent metabolic shift towards aerobic glycolysis
Source: Respir Res. 2019 May 9;20:87. doi: 10.1186/s12931-019-1058-2 (PMC6507142; doi:10.1186/s12931-019-1058-2)
Supplement: Supplementary file 1 — Supplementary Information (Supplementary Figures S1–S8). (DOCX 2671 kb) [file 12931_2019_1058_MOESM1_ESM.docx]

# Supplementary Information

**Human lung fibroblast-to-myofibroblast transformation is not driven by an LDH5-dependent metabolic shift towards aerobic glycolysis**

Eva Schruf, Victoria Schroeder, Christian A. Kuttruff, Sabine Weigle, Martin Krell, Maryke Benz, Tom Bretschneider, Alexander Holweg, Michael Schuler, Manfred Frick, Paul Nicklin, James P. Garnett*, Mirko C. Sobotta*

###
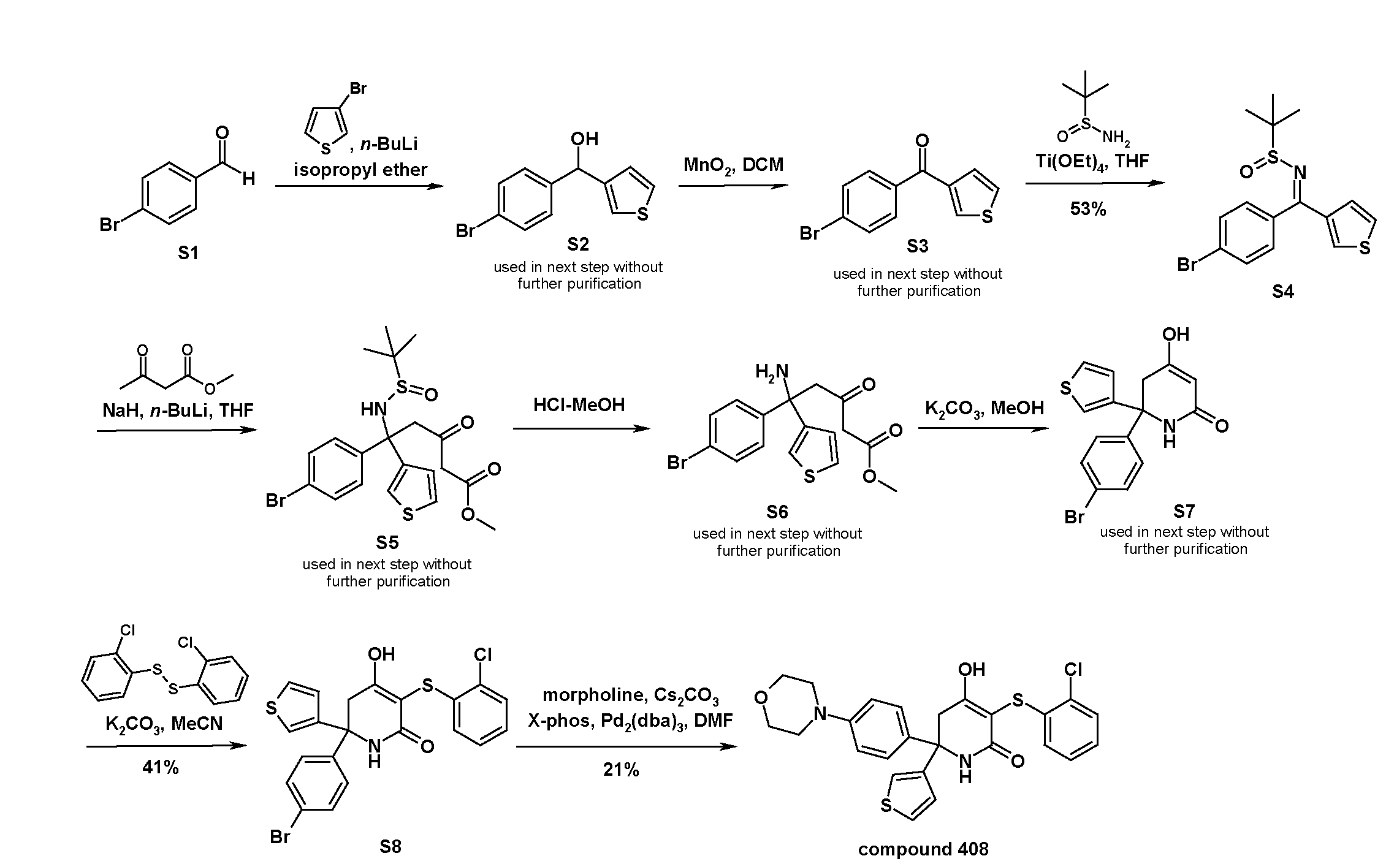


### Figure S1 – Synthesis of Compound 408. LDHA inhibitor Compound 408 was synthesized according to the procedures described in patent WO 2015/140133 A1. Purification of Compound 408: The final crude compound was purified by silica gel column chromatography (eluent: MeOH:dichloromethane = 1:100) to provide compound 408 as a colorless solid. Analytical Data for Compound 408: ^1^H-NMR (400 MHz, DMSO-d_6_) δ ppm 3.07 - 3.16 (m, 4 H) 3.39 (s, 2 H) 3.68 - 3.80 (m, 4 H) 5.92 (dd, *J*=8.05, 1.46 Hz, 1 H) 6.75 (ddd, *J*=7.90, 7.32, 1.24 Hz, 1 H) 6.93 (d, J=9.00 Hz, 1 H) 6.97 (ddd, *J*=7.98, 7.32, 1.46 Hz, 1 H) 7.15 (dd, *J*=5.09, 1.43 Hz, 1 H) 7.23 - 7.27 (m, 2 H) 7.28 (dd, *J*=7.94, 1.28 Hz, 1 H), 7.31 (dd, *J*=2.93, 1.46 Hz, 1 H) 7.56 (dd, *J*=5.05, 2.93 Hz, 1 H) 8.36 (s, 1 H) 11.39 (br s, 1 H); ^13^C-NMR (101 MHz, DMSO-d_6_) δ ppm 42.48 (s, 1 C) 48.22 (s, 2 C) 59.13 (s, 1 C) 66.05 (s, 2 C) 93.04 - 93.39 (range, 1 C) 114.50 (s, 2 C) 121.49 (s, 1 C), 124.83 (s, 1 C) 125.10 (s, 1 C) 126.50 (s, 1 C) 126.81 (s, 2 C) 126.91 (s, 1 C) 127.34 (s, 1 C) 128.70 (s, 1 C) 128.91 (s, 1 C) 134.98 (s, 1 C) 137.54 (s, 1 C), 146.74 (s, 1 C) 150.02 (s, 1 C) 166.28 (s, 1 C) 173.21 (s, 1 C); HRMS (ESI-TOF): calc’d for C_25_H_24_N_2_O_3_S_2_Cl [M+H]^+^ 499.0911, found: 499.0917.

###
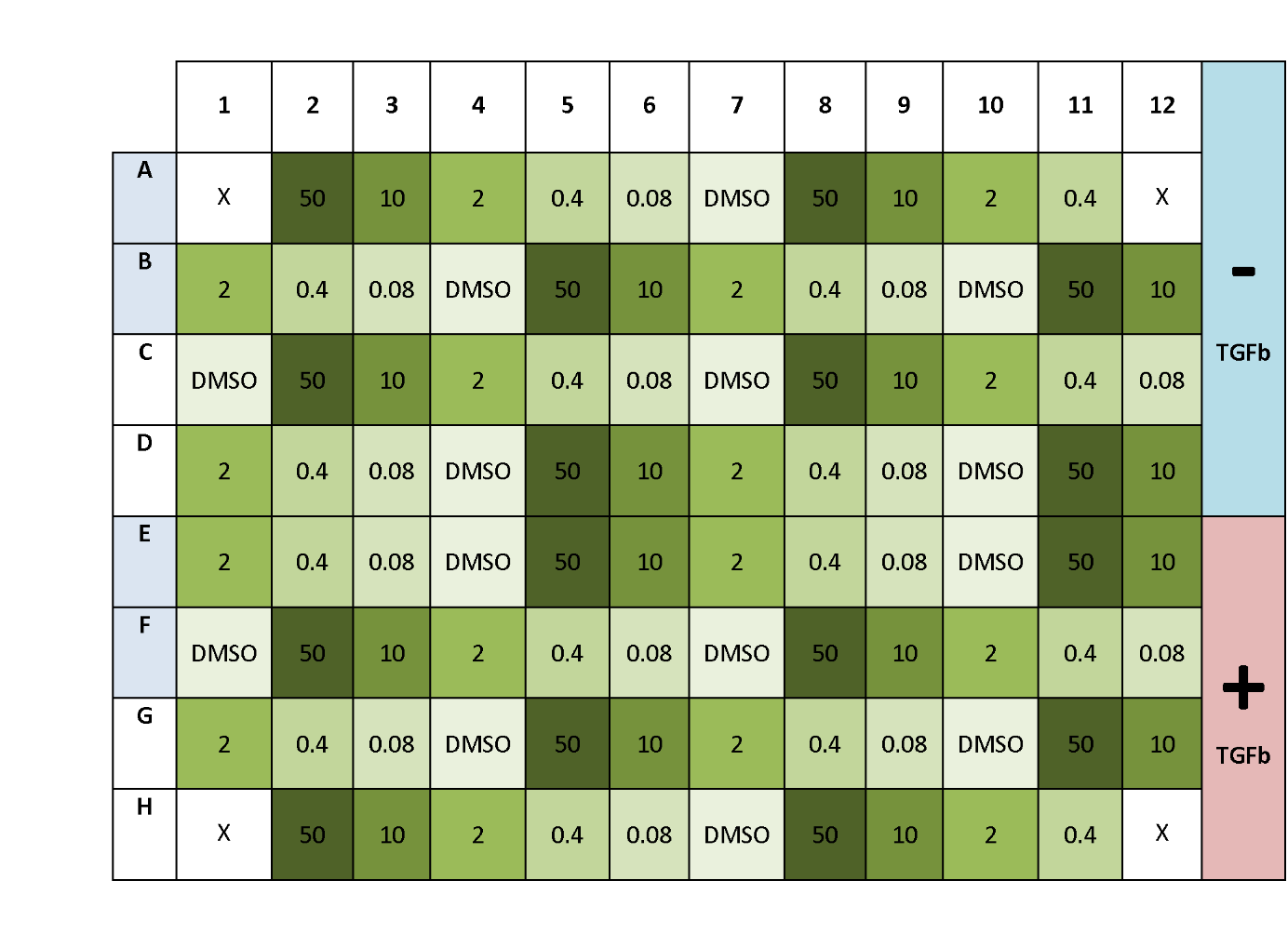


### Figure S2 – Example plate layout for Seahorse experiments. In order to minimize edge effects, a mirror-symmetrical plate layout was used that distributes samples treated with different inhibitor concentrations evenly on the plate. In general, the outer wells at all edges of the plate were seeded with cells and treated, but excluded for analysis, to further reduce edge effects.


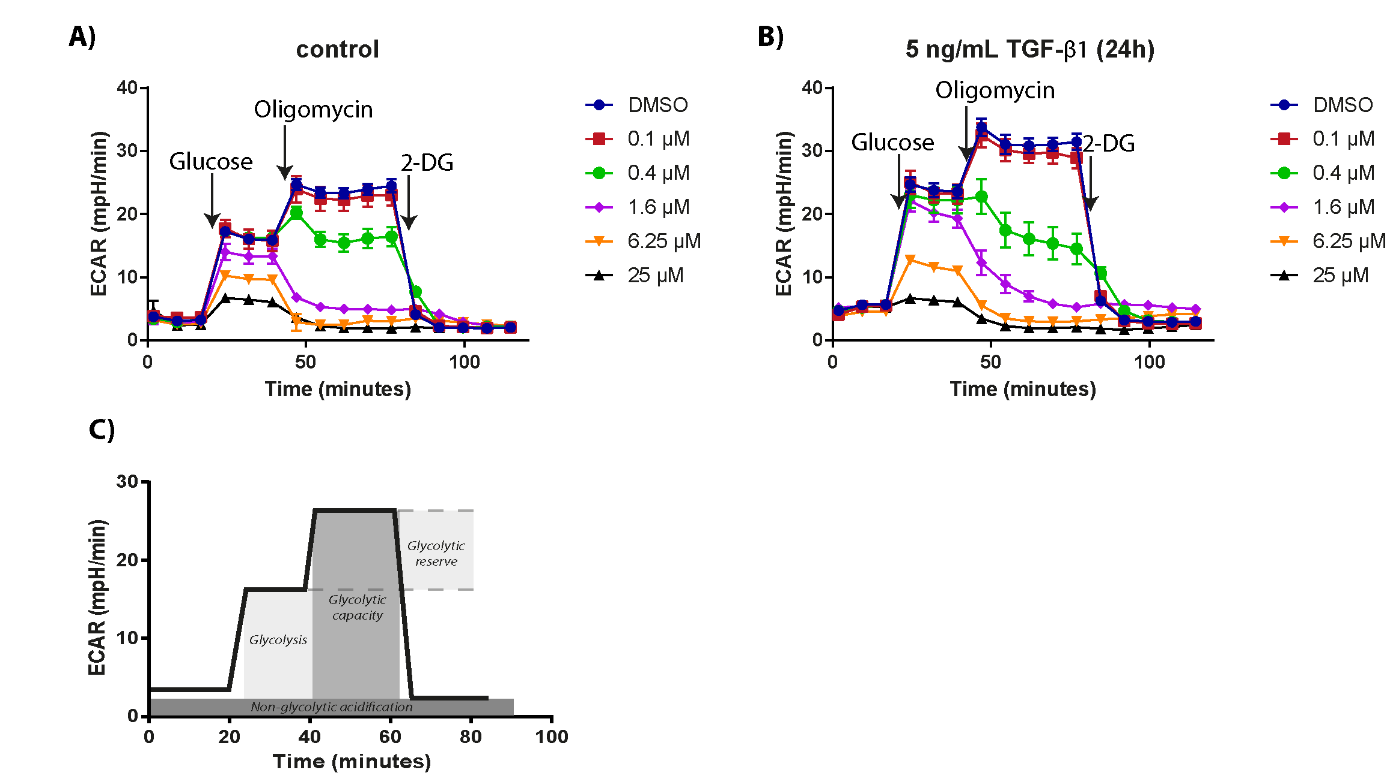


### Figure S3 – Raw data traces for dose-dependent inhibition of glycolysis by Compound 408 (data summarised in Figure 2). Extracellular acidification rate (ECAR) of WI-38 fibroblasts without pre-treatment (A) and with 24h pre-treatment with 5 ng/ mL TGF-β1 (B). During the glycolysis stress test assay cells were treated with 10 mM Glucose, 1.25 µM Oligomycin and 50 mM 2-Deoxyglucose (2-DG), to measure glycolysis, glycolytic capacity and glycolytic reserve (C). N=3.

###
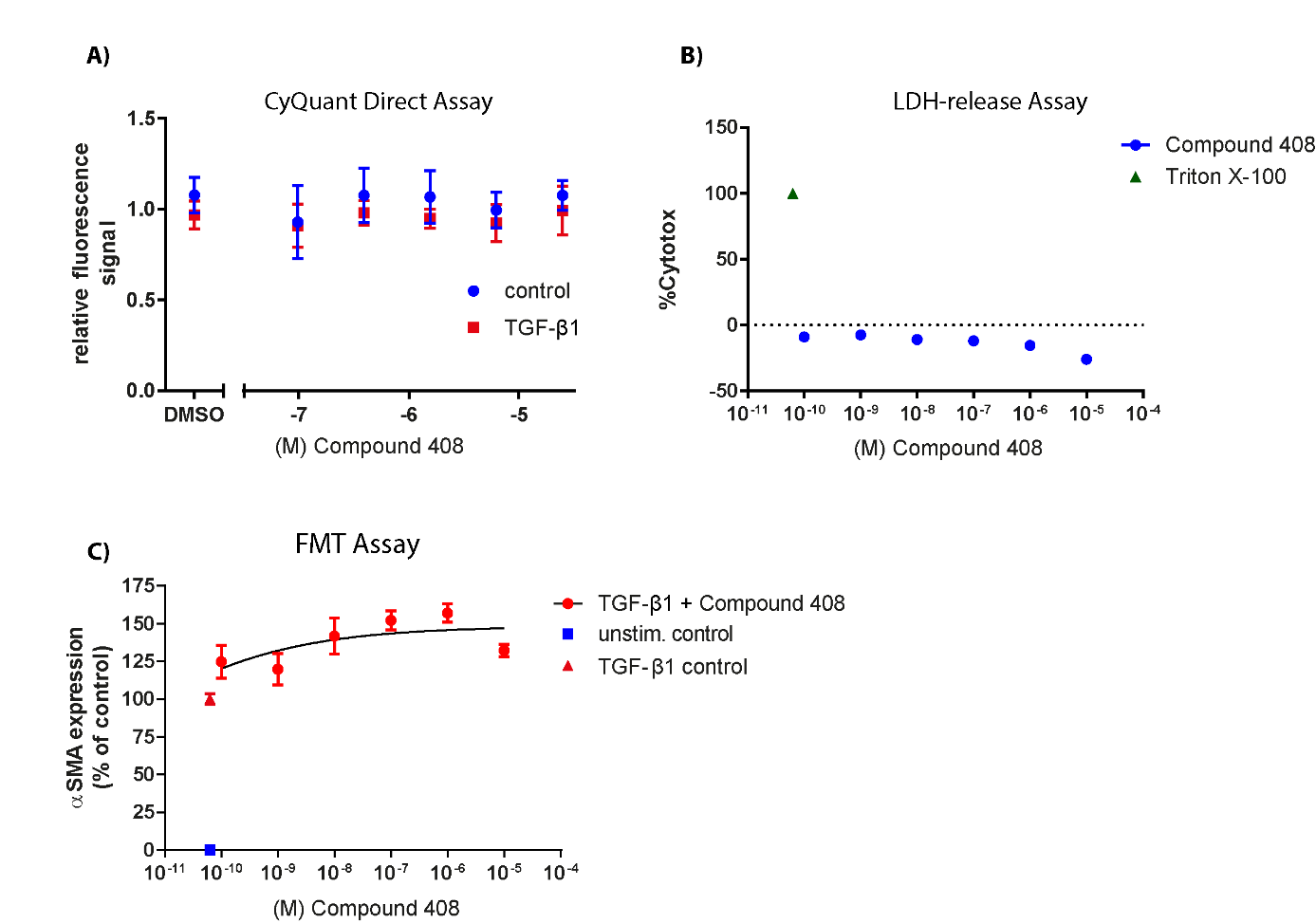


### Figure S4 – Compound 408 has no cytotoxic effects and does not inhibit FMT in NHLFs. Quantification of cytotoxicity induced in primary human lung fibroblasts by rising concentrations of Compound 408 by staining of nuclei of viable by CyQuant Direct Assay (A), and by LDH-release determined using a LDH Cytotoxicity Detection Kit ELISA (B). Effect of rising concentrations of Compound 408 on α-SMA protein expression measured via MSD ELISA, in TGF-β1 treated (5 ng/ mL) primary human lung fibroblasts (C). N=3.

###
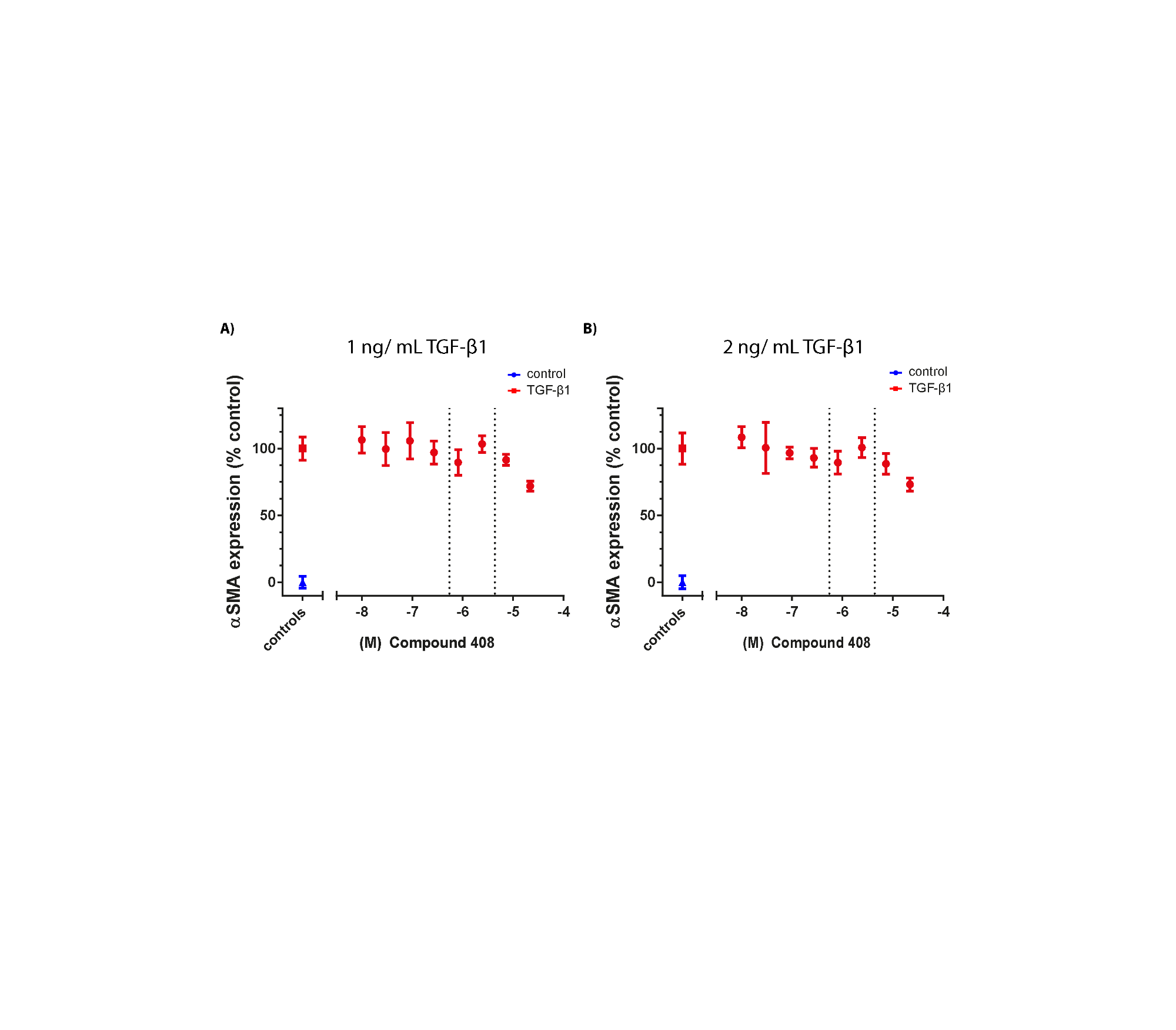


### Figure S5 – Compound 408 does not inhibit FMT in NHLFs under lowered TGF-β1 concentrations. Image based analysis of the effect of rising concentrations of Compound 408 on α-SMA expression (expressed as % of TGF-β1 treated control cells without compound) in primary human lung fibroblasts treated with either 1 ng/ml (A) or 2 ng/ml (B) TGF-β1 for 72h. N=2, n=8.

###
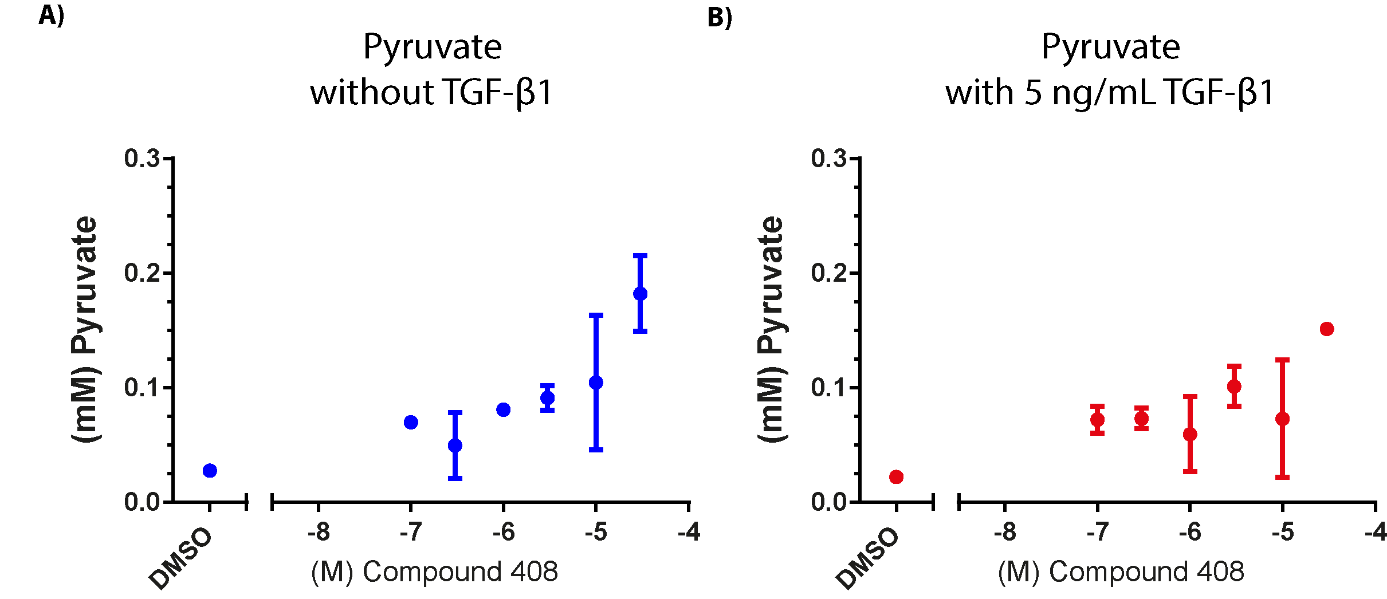


### Figure S6 – Compound 408 increases the extracellular pyruvate concentration of untreated and TGF-β1 treated NHLFs. The effect of rising concentrations of Compound 408 (72 h treatment) on pyruvate concentrations in the culture media of primary human lung fibroblasts without treatment (A) and with 72h treatment with 5 ng/ mL TGF-β1 (B). N=2, n=8.

###

###
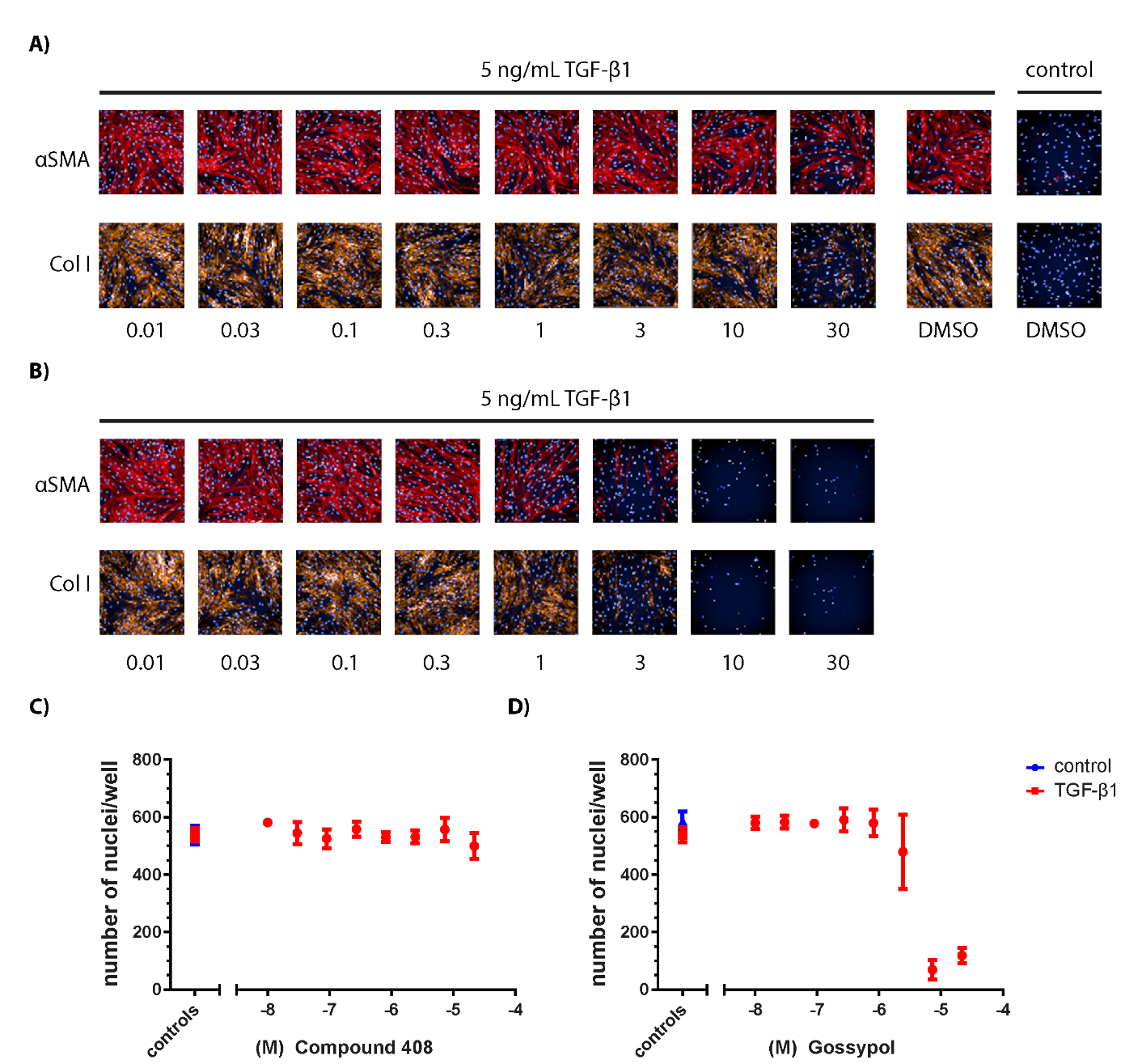


### Figure S7 – Decrease in FMT marker expression after Gossypol treatment of NHLFs is at least in part due to cytotoxic effects. Image analysis of the effects of LDH5 inhibitor (Compound 408) and Gossypol on α-SMA fibres, collagen I (A & B, respectively) and the numbers of nuclei (C & D, respectively) in TGF-β1-stimulated primary human lung fibroblasts after 72h. Compound 408 and Gossypol experiments run in parallel for direct comparison. N=2, n=8.


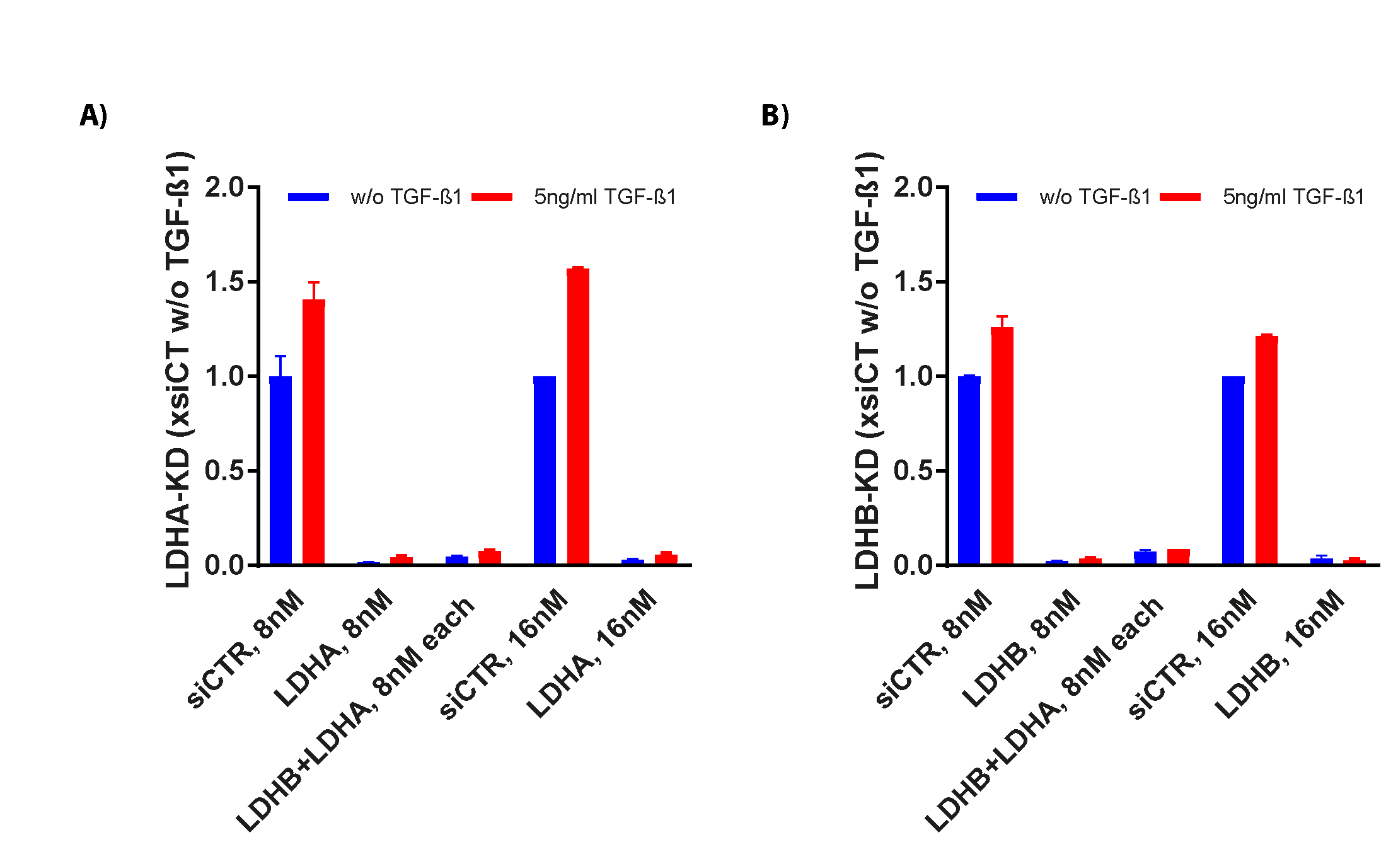


### Figure S8 – siRNA-mediated knockdown of LDHA and LDHB in NHLFs (related to Fig. 4). Primary human lung fibroblasts were treated with siRNAs targeting A) LDHA (LDH5) and B) LDHB followed by TGF-β1 stimulation. After 24 h, knockdown efficiency was confirmed by qRT-PCR. N=2, n=8.
